# Managing pneumonia through facility-based integrated management of childhood management (IMCI) services: an analysis of the service availability and readiness among public health facilities in Bangladesh

**DOI:** 10.1186/s12913-021-06659-y

**Published:** 2021-07-07

**Authors:** Ahmed Ehsanur Rahman, Shema Mhajabin, David Dockrell, Harish Nair, Shams El Arifeen, Harry Campbell

**Affiliations:** 1 University of Edinburgh, Edinburgh, UK; 2 International Centre for Diarrhoeal Disease Research, Dhaka, Bangladesh

**Keywords:** IMCI, Pneumonia, Service availability, Service readiness, And Bangladesh

## Abstract

**Background:**

With an estimated 24,000 deaths per year, pneumonia is the single largest cause of death among young children in Bangladesh, accounting for 18% of all under-5 deaths. The Government of Bangladesh adopted the WHO recommended Integrated Management of Childhood Illness (IMCI)-strategy in 1998 for outpatient management of pneumonia, which was scaled-up nationally by 2014. This paper reports the service availability and readiness related to IMCI-based pneumonia management in Bangladesh. We conducted a secondary analysis of the Bangladesh Health Facility Survey-2017, which was conducted with a nationally representative sample including all administrative divisions and types of health facilities. We limited our analysis to District Hospitals (DHs), Maternal and Child Welfare Centres (MCWCs), Upazila (sub-district) Health Complexes (UHCs), and Union Health and Family Welfare Centres (UH&FWCs), which are mandated to provide IMCI services. Readiness was reported based on 10 items identified by national experts as ‘essential’ for pneumonia management.

**Results:**

More than 90% of DHs and UHCs, and three-fourths of UH&FWCs and MCWCs provide IMCI-based pneumonia management services. Less than two-third of the staff had ever received IMCI-based pneumonia training. Only one-third of the facilities had a functional ARI timer or a watch able to record seconds on the day of the visit. Pulse oximetry was available in 27% of the district hospitals, 18% of the UHCs and none of the UH&FWCs. Although more than 80% of the facilities had amoxicillin syrup or dispersible tablets, only 16% had injectable gentamicin. IMCI service registers were not available in nearly one-third of the facilities and monthly reporting forms were not available in around 10% of the facilities. Only 18% of facilities had a high-readiness (score 8–10), whereas 20% had a low-readiness (score 0–4). The readiness was significantly poorer among rural and lower level facilities (*p* < 0.001). Seventy-two percent of the UHCs had availability of one of any of the four oxygen sources (oxygen concentrators, filled oxygen cylinder with flowmeter, filled oxygen cylinder without flowmeter, and oxygen distribution system) followed by DHs (66%) and MCWCs (59%).

**Conclusion:**

There are substantial gaps in the readiness related to IMCI-based pneumonia management in public health facilities in Bangladesh. Since pneumonia remains a major cause of child death nationally, Bangladesh should make a substantial effort in programme planning, implementation and monitoring to address these critical gaps to ensure better provision of essential care for children suffering from pneumonia.

**Supplementary Information:**

The online version contains supplementary material available at 10.1186/s12913-021-06659-y.

## Background

Bangladesh was one of the few high burden countries with limited resources which achieved the ambitious MDG-4 target of reducing the under-5 mortality rate by two-thirds) ahead of 2015 [1, 2]. Despite this commendable accomplishment, Bangladesh still suffers one of the highest under-5 mortality rates in the world [3]. According to the latest Demographic and Health Survey conducted in 2017, the under-5 mortality in Bangladesh was 45 per thousand live births, indicating an apparent stalling in the rate of reduction from the 2014 estimates of 46 per thousand live births [2, 3]. With an estimated 24,000 deaths per year, pneumonia accounted for 18% of all under-5 deaths, making it the single largest cause of death among young children [4]. Therefore, achieving the ambitious 2030-SDG target of reducing the under-5 mortality to 25 per thousand live births or below will require strategic focus, substantial investments and concerted efforts by all stakeholders by prioritisation of pneumonia at every stage [5, 6].

Cognizant of the high burden of pneumonia morbidity and mortality in the majority of Low- and Middle-Income Countries (LMICs), the World Health Organization (WHO) and the United Nations International Children’s Emergency Fund (UNICEF) set forth an ambitious goal to end all preventable deaths due to pneumonia by 2025 [5]. Bangladesh declared its commitment to reduce the pneumonia specific mortality rate among children less than 5 years of age from eight deaths per thousand live births to three deaths per thousand live birth by 2025 by implementing WHO and UNICEF’s Global Action Plan for Pneumonia and Diarrhoea (GAPPD) [5].

Ensuring timely and appropriate management of pneumonia through outpatient and inpatient care is one of the key components of GAPPD’s ‘prevent-protect-treat’ strategy [5]. For outpatient management of common childhood illnesses, including pneumonia, the global recommendation is to adopt the Integrated Management of Childhood Illness (IMCI) strategy in high-burden and low-resource settings [7, 8]. IMCI has demonstrated a positive impact in improving health workers’ performance and quality of care as well as reducing childhood mortality, including pneumonia in settings with limited resources [9–16]. However, success depends on the effective implementation of the three basic pillars outlined in the IMCI strategy, which include improving health workers’ skills, improving the health system to ensure supplies of essential items to provide quality services, and improving family and community practices [17–20]. Ensuring the service availability and readiness of health facilities are among the first steps towards strengthening the health systems [17, 18]. They are also fundamental to ensuring the provision of care, which is one of the core components of WHO’s Quality of Care Framework [21, 22].

The Government of Bangladesh (GoB) adopted the IMCI strategy in 1998, and facility-based IMCI was scaled up in all districts (64) and more than 90% of all sub-districts (420) by 2014 [16, 23]. According to the current programme implementation model, IMCI services are provided through a dedicated corner (i.e. IMCI corner) at the outpatient departments in all district and sub-district level hospitals [24, 25]. At the sub-district level, IMCI services are provided through union-level health centres. Doctors, nurses or paramedics (locally known as SACMOs) with special in-service training are responsible for providing IMCI services [24, 25]. Unfortunately, there is little evidence regarding the current status of service availability, readiness and quality of care rendered through IMCI services in Bangladesh. Such information, particularly those that are related to pneumonia management, is critical for understanding the health systems bottlenecks and taking course corrective measures to achieve the ambitious 2025-GAPPD and 2030-SDG targets.

This paper attempts to address this critical evidence gap by presenting the status of IMCI services, explicitly focusing on the service availability and readiness related to IMCI-based pneumonia management in Bangladesh, stratified by rural-urban, administrative division and type of health facilities.

## Method

### Data source

We conducted a secondary analysis of the Bangladesh Health Facility Survey (BHFS), which was conducted in 2017 with a nationally representative sample of health facilities [26]. The survey included all types of public hospitals and health centres as well as some private and NGO hospitals with at least 20 inpatient beds. The survey was carried out by the National Institute of Population Research and Training (NIPORT) with technical assistance from ICF International (USA) and the International Centre for Diarrhoeal Disease Research, Bangladesh (icddr,b). Data were collected by Associates for Community and Population Research (ACPR), Dhaka, a private research agency appointed by NIPORT for this survey.

### Study setting, sample size and outcome of interest

Bangladesh is divided into eight administrative divisions (Barisal, Chittagong, Dhaka, Khulna, Rajshahi, Rangpur, Sylhet, and Mymensingh), which are subdivided into 64 districts (zilas), and further divided into 485 sub-districts (upazilas). The public health systems in Bangladesh has three tiers of referral hospitals and two types of health centres. At the district level, there are Medical College Hospitals as tertiary level referral hospitals, District Hospitals (DHs) as secondary level referral hospitals with 250–500 beds. At the sub-district level, there are Upazila Health Complexes (UHCs) as primary level referral hospitals with 50 beds. Below the sub-district level, there are Union Health and Family Welfare Centres (UH&FWCs) and Community clinics (CCs) and) as health centres. In addition, there are Maternal and Child Welfare Centres (MCWCs) in all districts and few sub-districts with 10 beds. DHs have a catchment population of around 2–3 million, while it is 250,000–300,000 for UHCs, 25,000–30,000 for UH&FWCs and 6000–10,000 for CCs.

The survey adopted a stratified random sampling procedure where the health facilities were stratified according to their administrative units and type of facilities. All divisions and the following types of health facilities were included in the survey: DHs, MCWCs, UHCs, UH&FWCs, CCs, and private hospitals and NGO clinics with at least 20 beds. In BHFS-2017, data were successfully collected from 1524 health facilities (95% response rate). From each facility, an average of eight health care providers, who provided the range of services being assessed, were selected for interviews. In facilities with less than eight health care providers, all providers present on the day of the visit were interviewed. A total of 5400 providers were interviewed [26].

### Data collection tools

BHFS 2017 used two types of questionnaires for data collection: a facility inventory questionnaire and a health care provider interview questionnaire. The facility inventory questionnaire was used to collect data related to the service availability and readiness of each priority services. The health care provider interview questionnaire was used to collect information related to the level of education, training, clinical experience, and supervision received by a sample of health care providers from each facility.

### Training and data collection

The data collection team consisted of 40 medical doctors and 40 paramedics who received three weeks of training from 09 July to 27 July 2017 in Dhaka. Data were collected between 30 July and 19 October 2017. Additional details are available in the BHFS-2017 report [26].

### Data analysis plan

Data were analysed using Stata version 14 (StataCorp, College Station, TX).

According to the national IMCI programme, all DHs, MCWCs, UHCs and UH&FWCs are mandated to provide facility-based IMCI services through outpatient departments [24, 25]. Therefore, in this paper, we limited our analysis to DHs (*n* = 62), UHCs (*n* = 141), UH&FWCs (*n* = 677), and MCWCs (*n* = 90), which were consistently used as denominators throughout the analysis.

**Fig. 1:**
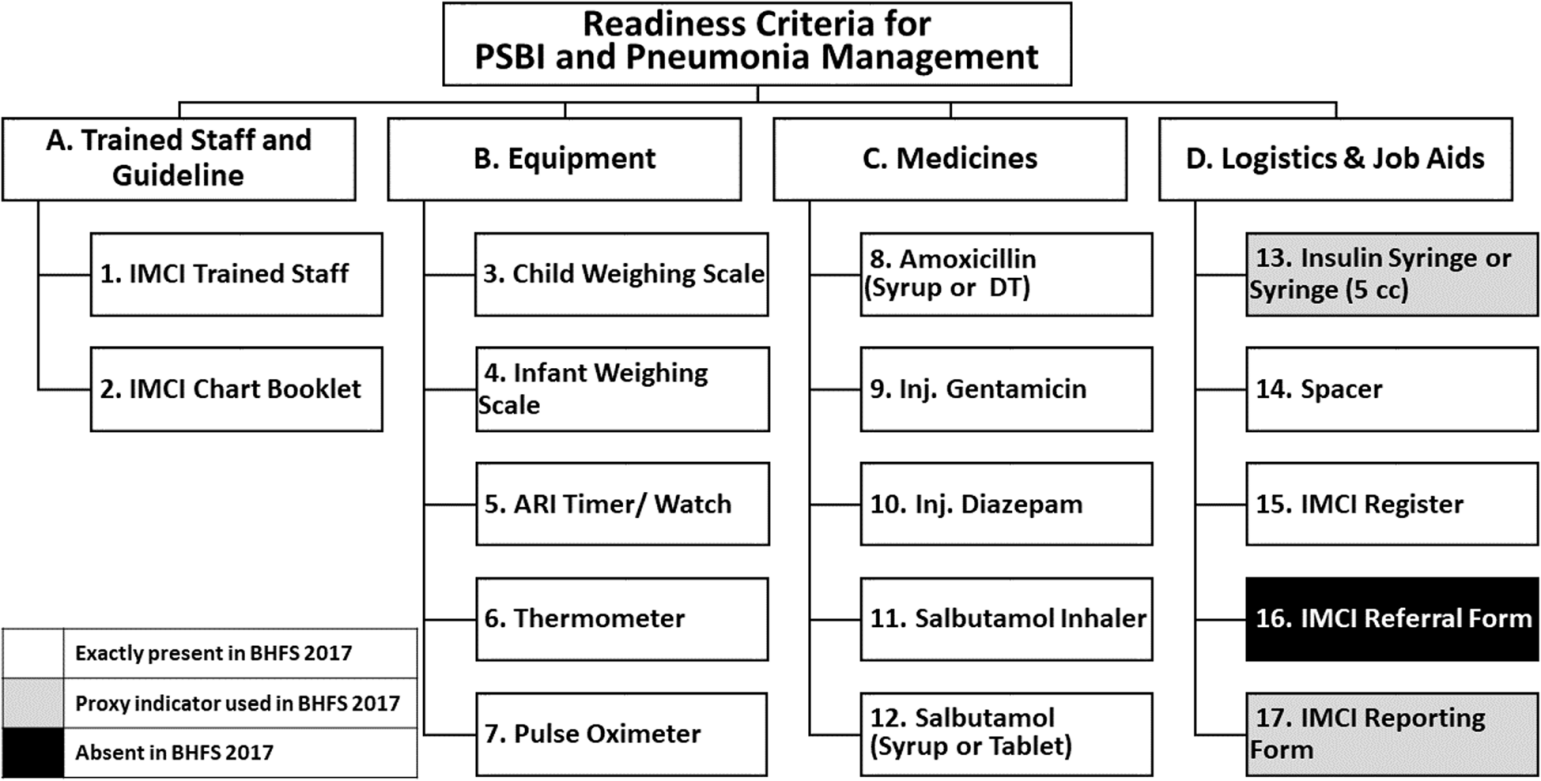
Items required for managing pneumonia based on facility-based IMCI Guidelines

The National Newborn Health Programme and IMCI (NNHP&IMCI) of Directorate General of Health Services (DGHS), Ministry of Health and Family Welfare (MoH&FW), Bangladesh has an approved list of essential equipment, drugs and logistics for IMCI services in Bangladesh. A consultative workshop was organised with the national experts under the leadership of IMCI Programme to review the IMCI-list and identify items ‘required’ for pneumonia management based on the facility-basedIMCI guidelines. The national experts identified 17 items as ‘required’, which was categorised into four domains: A. staff, B. equipment, C. medicines and D. logistics & job aids. Out of these 17 items, 14 were available, and three were absent in the BHFS-2017 tool. However, we identified proxy items (disposable syringe instead of Insulin syringe or 5 cc syringe; monthly reporting form instead of IMCI reporting form) in the tool for two of the absent items. The national experts also identified 10 items from the list of 17 required items as essential for managing pneumonia based on facility-based IMCI guidelines. Supplementary Table 2 presents the list of essential items. A ten-point scoring system was developed to measure the minimum pneumonia management readiness of designated health facilities. The availability of each of the essential item was given an equal score of one. Then, the facilities were categorised as ‘poor readiness: score 0–4, ‘moderate readiness: score 5–7 and ‘high readiness: score 8–10. The scoring system was developed in consultation with national experts and IMCI programme management team. Figure 1 summarises the items identified by the Technical Committee and their availability in BHFS 2017 tool.

Service availability was defined as facilities offering curative child care and reporting to provide the service based on facility-based IMCI guidelines. Readiness was defined as the presence of required items listed in Fig. 1 on the day of the visit. Supplementary Table 1 present the operational definition of readiness for each of the required items. Descriptive statistics (proportions) were used to report availability and readiness for each item. The estimates were stratified by facility type (DHs, UHCs, UH&FWCs, MCWCs), location (rural and urban) and administrative division.

In addition to the required and essential items, we present the readiness of IMCI facilities regarding the oxygen system. In addition to pulse oximetry (which was already included as a required item), the following items were included for presenting oxygen system readiness: availability of oxygen concentrator, availability of oxygen cylinder with flow meter, availability of oxygen cylinder without flow meter, and availability of oxygen distribution system.

## Results

Table 1 describes the background characteristics of the public health facilities, which are mandated to provide facility-based IMCI services in Bangladesh and included in this analysis. All of the DHs and the majority (79%) of the MCWCs were situated in urban areas. Regarding UHCs, around half were located in urban areas. In contrasts, almost all the UH&FWCs were located in rural areas. Among all the facilities, around one-fifth were in Barisal and Chittagong divisions each while rest of the divisions, contributed around 10% each.

**Table 1:** Background characteristics of the facilities mandated to provide facility-based IMCI services in Bangladesh, presented in frequency and column percentage

	DH		UHC		UH&FWC		MCWC		Total	
	n	%	n	%	n	%	n	%	n	%
**Location**										
Urban	62	100%	77	55%	6	1%	71	79%	216	22%
Rural	0	0%	64	45%	671	99%	19	21%	754	78%
**Division**										
Barisal	6	10%	26	18%	141	21%	10	11%	183	19%
Chittagong	11	18%	25	18%	151	22%	18	20%	205	21%
Dhaka	14	23%	15	11%	67	10%	14	16%	110	11%
Khulna	10	16%	14	10%	57	8%	13	14%	94	10%
Rajshahi	7	11%	17	12%	61	9%	13	14%	98	10%
Rangpur	7	11%	14	10%	58	9%	12	13%	91	9%
Sylhet	4	6%	18	13%	90	13%	6	7%	118	12%
Mymensingh	3	5%	12	9%	52	8%	4	4%	71	7%
**Total**	62	100%	141	100%	677	100%	90	100%	970	100%

**Fig. 2:**
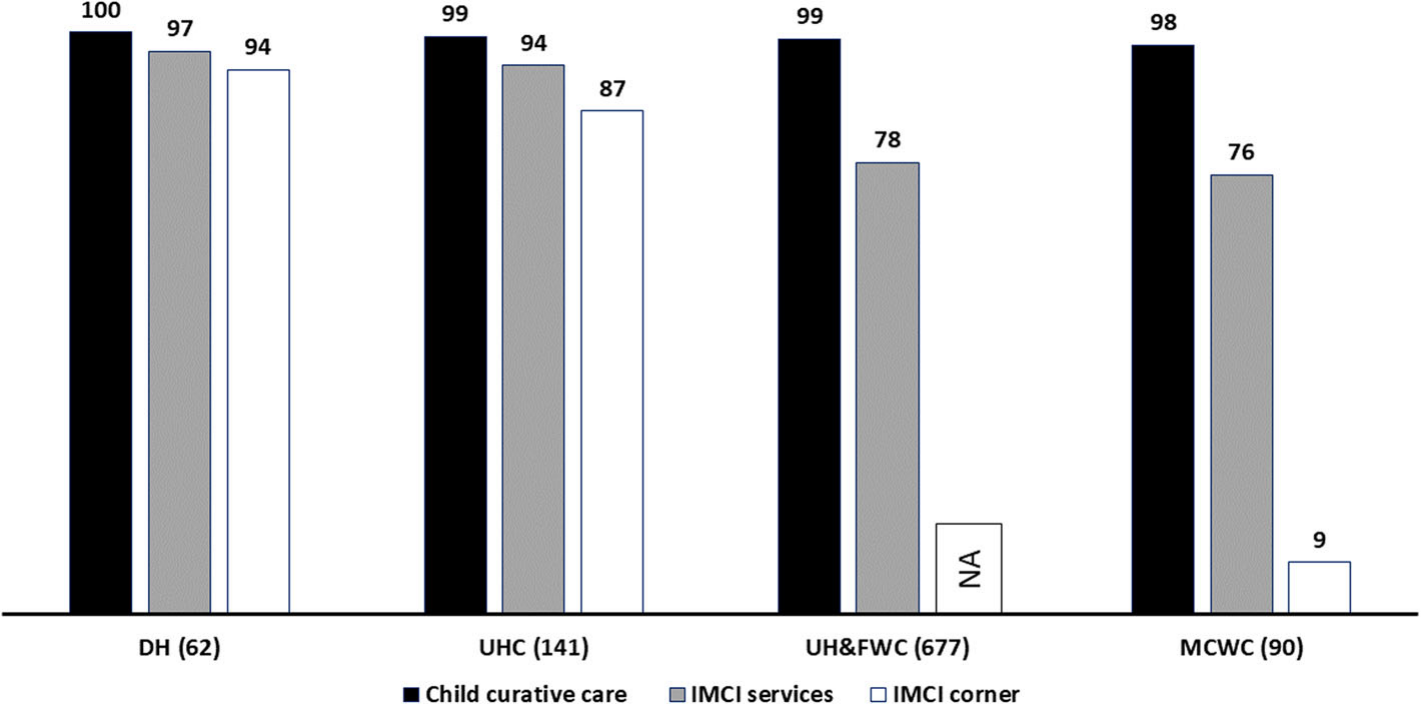
Service availability related to IMCI services (including pneumonia management) in different types of public health facilities mandated to provide facility-based IMCI services in Bangladesh, presented in percentage (N = 970)

Figure 2 summarises the service availability related to pneumonia management in public health facilities mandated to provide facility-based IMCI services in Bangladesh. Curative child care is almost universally available across all types of facilities. More than 90% of DHs and UHCs reported providing IMCI services (including pneumonia management), whereas such services were available in three-fourths of UH&FWCs and MCWCs. Around 90% of DHs and UHCs had IMCI corners. However, the availability was extremely low (9%) in MCWCs. UH&FWCs were not mandated to establish specific corners for providing IMCI services; therefore the findings for this facility have been presented as ‘not applicable’.

**Fig. 3:**
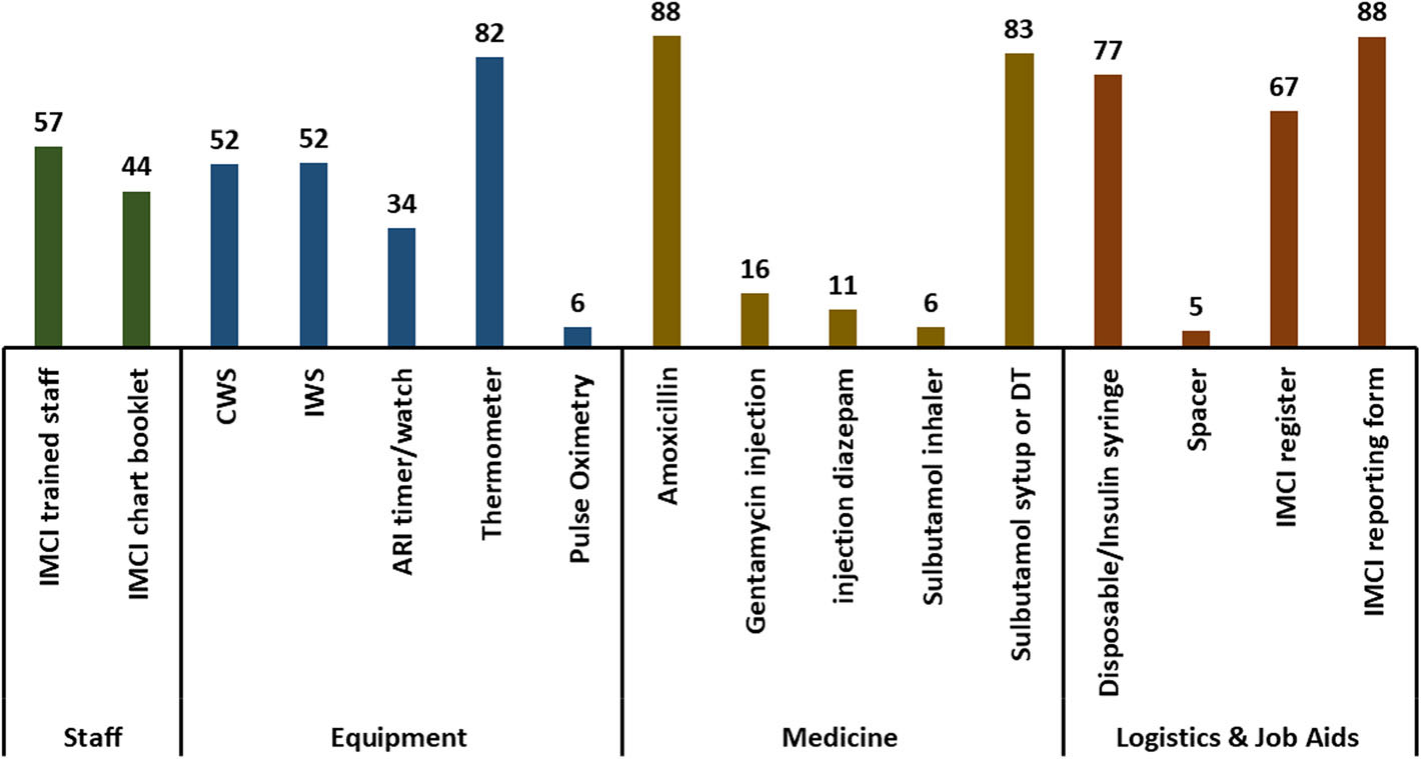
Availability of the items required for managing pneumonia in public health facilities mandated to provide facility-based IMCI services in Bangladesh, presented in percentages (*N* = 970)

Figure 3 illustrates the readiness related to the management of pneumonia in different types of public health facilities mandated to provide facility-based IMCI services in Bangladesh. The availability (on the day of the visit) is presented for each of the items (presented in Fig. 1) required for managing pneumonia according to facility-based IMCI guidelines. Around 57% of the facilities had a staff ever trained in facility-based IMCI guidelines, and 44% had a copy of the IMCI chart booklet on the day of the visit. Regarding equipment, the thermometer was available in 82% of facilities. Weighing scales (child or infant) were available in half of the facilities. Only one-third of the facilities had an ARI timer or a watch that can record time in seconds on the day of the visit. Pulse oximetry was available in only 6% of facilities. More than 80% of the facilities had amoxicillin syrup or dispersible tablets on the day of the visit. However, the availability of injection gentamicin was only 16%. An Insulin syringe or disposable 5 cc syringe was available in 77% of facilities. Although monthly reporting forms were available in 88% of facilities, around one-third of the facilities did not have an IMCI service register to document their care practices.

Table 2 presents the readiness (availability of required items on the day of the visit) related to the management of pneumonia in public health facilities mandated for providing facility-based IMCI services in Bangladesh, disaggregated by facility type, location and division for each of the readiness items. DHs and UHCs demonstrated higher levels of readiness than UH&FWCs for the majority of the items. The readiness of UH&FWCs was particularly low (less than half of DHs) for IMCI chart booklet, weighing scale, ARI timer, pulse oximeter, injection gentamicin, injection diazepam and IMCI register. Similarly, the readiness was higher among urban-facilities than that of rural-facilities across all items. Pulse oximetry was available in only 27% of the district hospitals and 18% of the UHCs. None of the UH&FWCs had a pulse oximeter on the day of the visit. No obvious pattern was observed regarding the readiness across different divisions. More than two-thirds of UHCs had both IMCI trained staff and guideline, but around a quarter of MCWCs and union facilities had both. Similar gaps were identified of all other domains.

**Table 2:** Availability of the items required for managing pneumonia in public health facilities mandated to provide facility-based IMCI services in Bangladesh, presented in percentages by facility type, location and division

	Staff and guideline			Equipment						Medicines						Logistics				
	1. IMCI TS	2. IMCI CB	All	3. CWS	4. IWS	5. ARI or TW	6. Th	7. PO	All	8. Amox	9. Inj. G	10. Inj. D	11. SI	12. SS/ST	All	13. IS	14. Sp	15. IMCI R	16. IMCI RF	All
**Type**																				
DH	79	69	53	71	69	45	87	27	11	94	44	61	31	92	11	73	23	94	97	16
UHC	85	72	63	73	69	46	92	18	5	89	41	31	17	92	4	75	17	89	97	14
Union level public facilities	57	37	24	45	48	29	79	0	0	87	8	1	2	81	0	77	1	61	87	0
MCWC	59	37	23	57	61	47	86	17	3	93	10	23	1	81	0	83	7	59	79	2
**Location**																				
Urban	78	61	49	69	66	49	90	22	7	92	34	40	17	89	5	76	15	80	90	11
Rural	58	40	27	47	50	30	80	1	0	87	10	3	3	81	0	77	2	63	87	1
**Division**																				
Barisal	72	51	37	50	59	36	89	6	2	85	19	8	4	79	1	81	4	70	91	3
Chittagong	56	41	29	49	54	39	84	4	2	89	23	8	3	91	2	78	5	58	89	2
Dhaka	68	48	35	43	30	29	71	6	0	82	10	16	15	71	2	75	6	69	91	5
Khulna	64	40	31	59	60	31	78	11	3	92	16	16	7	81	0	76	5	70	87	4
Rajshahi	58	35	25	40	57	37	87	7	1	89	11	14	7	83	2	79	6	75	91	6
Rangpur	62	44	34	62	62	30	82	7	1	93	8	17	4	87	3	67	6	69	82	4
Sylhet	61	42	27	63	55	38	86	5	3	89	14	8	5	85	2	88	5	63	80	2
Mymensingh	59	54	37	58	48	21	68	3	0	90	10	7	4	85	0	61	1	70	90	1

1.IMCI TS: IMCI trained staff

2. IMCI CB: IMCI chart booklet

3. CWS: Child weighing scale

4. IWS: Infant weighing scale

5. ARI TW: ARI Timer or watch able to record seconds hand

6. TH: Thermometer

7. PO: Pulse oximeter

8. Amox: Amoxicillin Dispersible Tablets or syrup 9. Inj. G: Injection Gentamicin

10. Inj. D: Injection Diazepam

11. SI: Salbutamol inhaler

12. SS/ST: Salbutamol syrup/tablet

13. IS: Insulin syringe or disposable 5 cc syringe

14. S: Spacers

15. IMCI R: IMCI registers

16. IMCI RF: IMCI reporting form

**Fig. 4:**
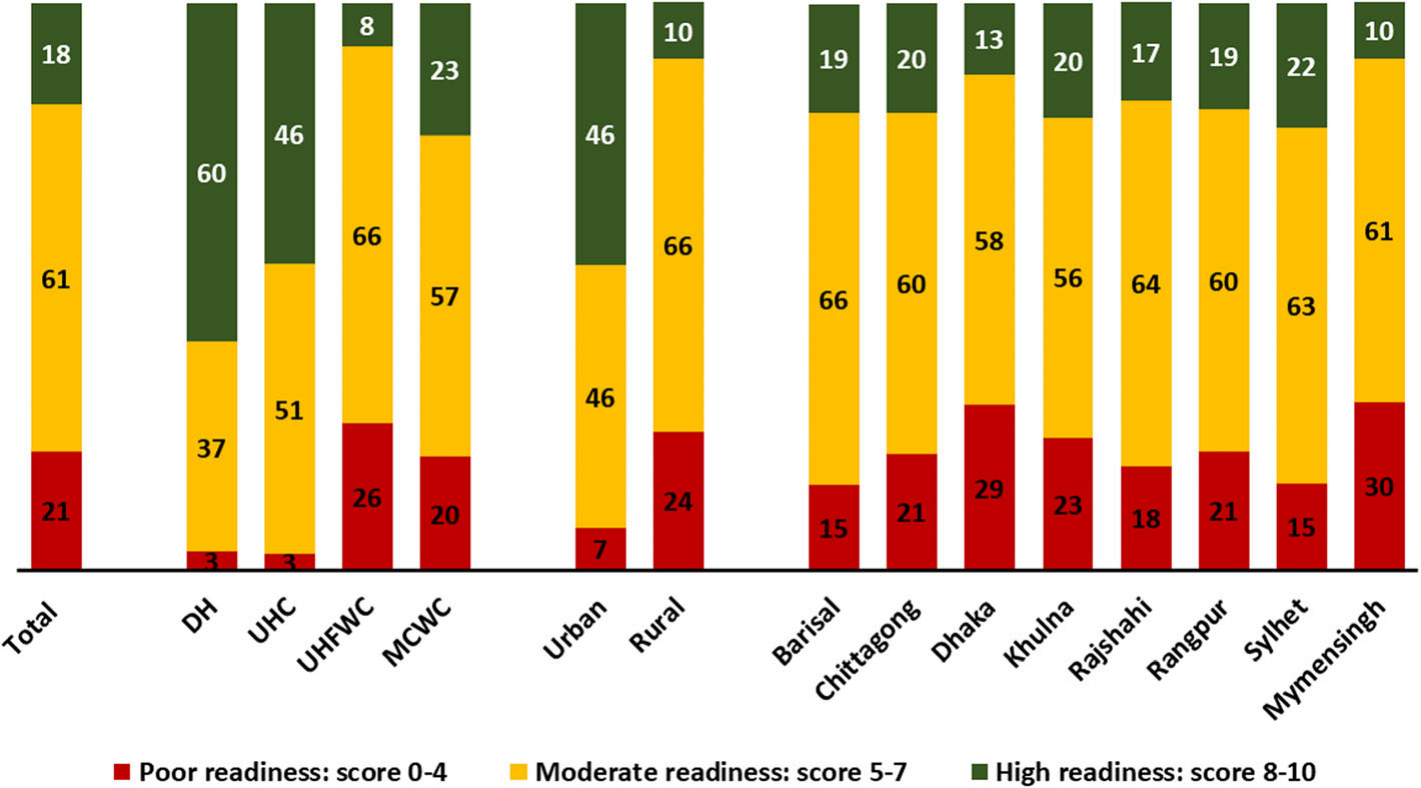
Availability of items essential for managing pneumonia in public health facilities mandated to provide facility-based IMCI services in Bangladesh, presented in percentages based on a ten-point readiness score, by facility type, location and division

Figure 4 presents the readiness based on ten essential items for managing pneumonia in public health facilities mandated to provide facility-based IMCI services in Bangladesh. The score denotes the number of items available on the day of the visit. Out of the facilities surveyed, only 18% had a high level of readiness with an overall readiness score of 8–10, whereas 20% had a low level of readiness with an overall score of 0–4. When stratified by facility type, DH and UHC showed a higher level of readiness than that of UH&FWCs (60% for DHs, 46% for UHCs and 8% for UH&FWCs, *p* < 0.001). Facilities located in urban areas had a much higher level of high readiness (46%) than facilities located in rural areas (10%) (p < 0.001). The readiness patterns were similar across all administrative divisions. Supplementary Figure 1 illustrates that no facilities had all of the ten essential items available on the day of the visit. Around 5% of the DHs and UHCs had all essential items, whereas only 1% of MCWCs and no union facilities had all essential items.

**Fig. 5:**
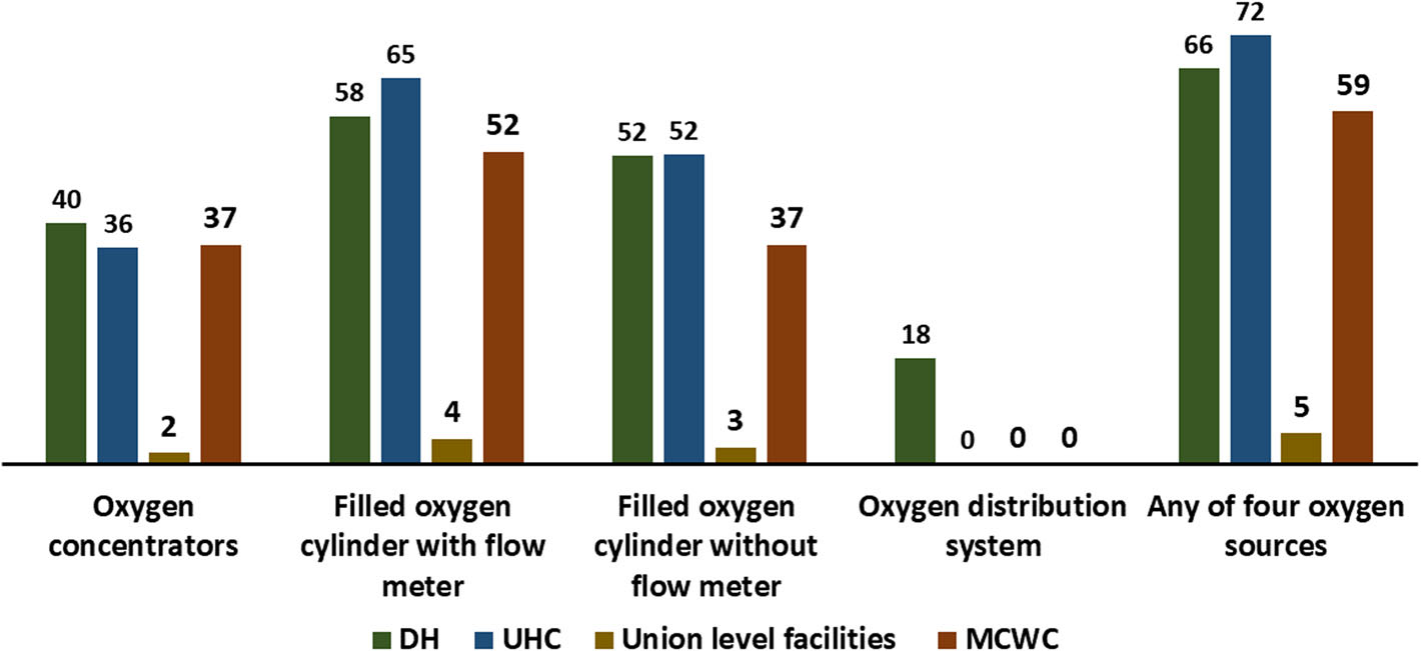
Availability of oxygen sources in different types of public facilities in Bangladesh, presented in percentages

Figure 5 illustrates the readiness related to oxygen sources in public health facilities in Bangladesh. Around 40% of the DHs and one-thirds of the UHCs and MCWCs had a functioning oxygen concentrator on the day of the visit. More than half (52–65%) of the DHs, UHCs and MCWCs had oxygen cylinders with flow meters. Besides, around half of the DHs and UHCs, and one-third of the MCWCs had oxygen cylinders without flowmeters on the day of the visit. Around 18% of the district hospitals had an oxygen distribution system, but none of UHCs, UH&FWC and MCWCs had this. The availability of any of the four oxygen sources was the highest in UHCs (72%) followed by DHs (66%) and MCWCs (59%). Oxygen availability was particularly low (5%) in UH&FWCs.

## Discussion

Pneumonia is the largest killer of children under five years of age in Bangladesh, and IMCI is one of the key strategies adopted by the Government of Bangladesh for managing pneumonia [3, 24, 25]. The success of IMCI services will greatly influence Bangladesh’s progress towards achieving the GAPPD target of preventing all avoidable pneumonia-related deaths by 2025 [5]. Ensuring the service availability and readiness of health facilities are the stepping stones for health systems strengthening and critical to promoting the quality of care rendered through IMCI services [27]. This is the first study to report the service availability and readiness related to IMCI-based pneumonia services in Bangladesh through a nationally representative sample. This study identified that there were substantial gaps in staff trained in IMCI services, availability of essential items like functional ARI timer or watch that can record time in seconds, pulse oximeter, registers and reporting forms for documentation, and necessary medicines for providing IMCI-based pneumonia management. Moreover, there were critical gaps in the availability of oxygen sources in the surveyed facilities.

WHO developed the IMCI strategy in the mid-1990s, which incorporated key lessons from WHO’s Global Acute Respiratory Infection Control Programme launched in 1980s [27]. The Government of Bangladesh adopted the IMCI strategy immediately after that, and the process of health systems integration and national scale-up were completed through its inclusion in the National Health Sector Programme 2011–16 and Essential Service Package [28, 29]. According to the current National Health Sector Programme 2017–22, all types of public facilities (DH, UHCs, UH&FWCs and MCWCs) are mandated to provide curative child care through IMCI services [24, 25]. Our analysis reveals that almost all facilities offer child curative services, but there are substantial gaps regarding the availability of IMCI services in UH&FWCs and MCWCs. The divide between the two directorates of the Ministry of Health and the differences in their strategic focus can potentially explain such contrasting pictures. All DHs and UHCs are under the DGHS, however, the majority of the UH&FWCs and all MCWCs are under the Directorate General of Family Planning (DGFP) [24, 25]. Historically, DGHS was involved in the introduction and scale-up of IMCI services in Bangladesh, and they have a specific programme named after IMCI (NNHP&IMCI). On the other hand, the prime mandate of DGFP is to promote and provide family planning services. Child curative care was incorporated into DGFP’s mandate as an expansion of the existing family planning services. Also, there are gaps in coordination between the IMCI programme of DGHS and the child health programme of DGHS.

According to the current health sector programme, all DHs, UHCs and MCWCs are supposed to prioritise IMCI services by maintaining a dedicated corner in the outpatient department [24, 25]. IMCI corners require dedicated staff (IMCI trained), equipment and logistics, which helps operationalising outpatient services and retaining the skills of the service providers. The IMCI corners also help in managing the high-volume patient flow in DHs, UHCs and MCWCs, and act as a referral link for children who are referred from UH&FWCs. Contrary to the policy outlined in the health sector programme, 6% of DHs, 13% of UHCs, and more than 90% of MCWCs do not have dedicated IMCI corners. The lack of a strategic focus and programmatic investments by DGFP regarding IMCI services can potentially explain the low availability of IMCI corners in MCWCs. In addition to the IMCI corners, different programmes have recommended maintaining various other corners like ANC corner, PNC corner, KMC corner, SCANU corner, NCD Corner, Breast Feeding corner, VIA corner etc. in DHs and UHCs [24, 25, 29]. The competing interests of various corners can, which were established as a horizontal integration of various services, be another explanation for the gaps in the availability of IMCI corners in facilities with limited resources.

IMCI adopts a syndromic management approach, where the service provider has to follow various clinical algorithms to classify and treat childhood illnesses [30]. It requires continuous in-service training and supportive supervision to retain the knowledge and skills of the service provider and ensure the quality of IMCI services [20]. Unfortunately, almost half of the surveyed facilities do not have an IMCI trained staff and a copy of the chart booklet. The situation is particularly worse in UH&FWCs and MCWCs. Although DHs and UHCs demonstrate a comparatively higher level of readiness regarding the availability of a service provider ever trained in IMCI only around 31% of the DHs and approximately one-third of the UHCs have a service provider who received IMCI training within the past 24 months [26]. It raises a critical concern regarding the quality of IMCI services, including management of pneumonia rendered through the service provider without appropriate training and supervision. It reveals the apparent gaps in national programme planning and implementation.

The survey (BHFS) was conducted in 2017, which is just after the completion of the previous National Health Sector Programme 2011–16 [26, 28]. IMCI training organised by the IMCI programme in the last sector programme implementation period was not adequate to the needs of the country. Since 2017, the Ministry of Health is implementing the current National Health Sector Programme 2011–16 [31]. The provision and resource allocation for IMCI training in the operation plan of the current health sectors programme is also inadequate and insufficient [24, 25]. Moreover, in the first two years of the current sector programme implementation period (2017–19), the IMCI programme did not organise any IMCI training for the old and newly recruited service providers. One of the core components of IMCI strategy is to improve the case-management skills of service providers through the provision of locally adapted IMCI-guidelines and through activities to promote their use. Unfortunately, more than half of the surveyed facilities do not have a copy of the chart booklet. Moreover, the latest version of Bangladesh adapted version of the IMCI chart booklet is based on WHO’s 2008-edition [32, 33]. In 2014, WHO released another version of IMCI chart booklet with major updates regarding pneumonia classification, treatment recommendations and referral criteria [30, 34]. During the implementation periods of the previous health sector programme (2011–16) and the first two years of the current health sector programme (2017–22), the IMCI programme could not take proper initiatives to update the IMCI chart booklet based on WHO’s recent recommendations as well as print and distribute them to the IMCI service delivery points in DHs, UHCs, UH&FWCs and MCWCs. This could substantially affect the safety of treatment and quality of care, particularly for pneumonia and sepsis management.

IMCI is designed for settings with minimum diagnostic capacity. Therefore, the availability of some basic equipment and medicine must be ensured for proper clinical assessment and treatment according to the guidelines [30]. Assessment of respiratory rate is one of the most important steps in IMCI-based pneumonia classification, which eventually determines the management plan, referral decision and treatment outcomes [34–36]. Unfortunately, two-thirds of the designated facilities in Bangladesh do not have a functional ARI timer or watch that is able to record time in seconds. Moreover, around half of the facilities do not have an appropriate weighing scale which is important for prescribing medicines (including antibiotics of pneumonia management) with appropriate dosages [30]. The majority of these essential equipment are not costly and are readily available in the local market. Therefore, the observed gaps in their availability reflect the apparent lack of effective programme planning, coordination and monitoring.

The IMCI-based pneumonia management requires the availability of oral amoxicillin and injectable gentamicin as the first-line treatment for severe pneumonia and possible serious bacterial infection of young infants [37, 38]. For more severe cases, the availability of both amoxicillin and gentamicin together is essential for prereferral treatment and outpatient-based management (when applicable) [39]. Although the availability of oral amoxicillin was reasonably high among all types of facilities, there are substantial gaps regarding the availability of injectable gentamicin. Such critical gaps in readiness regarding the first-line antibiotics essentially reduce the level of effective coverage, may compromise the level of adherence to guidelines and potentially result in avertable pneumonia-related deaths [40]. Besides the availability of the IMCI medicines, the choice of antibiotics recommendations by IMCI services providers may be influenced by external factors. Several studies conducted in Bangladesh reported that there are substantial gaps regarding health care providers’ adherence to guidelines since many of them prefer higher general antibiotics over the first-line recommended antiiotics [41–44].

Documentation is one of the most important parts of IMCI services. The IMCI service register is designed to capture essential details on clinical assessments, disease classifications, treatments, and referral decisions. It acts as a job aid to assist the provider in adhering to the guideline and prepare monthly reports efficiently [45]. Registers are also linked to the overall monitoring and supervision framework laid out for IMCI services, as well as the overall accountability structure [20, 45]. Although registers are almost universally available in DHs and UHCs, one-third of UH&FWCs and MCWCs do not have them, according to our analysis. The lack of availability of IMCI registers also raises questions regarding the validity of data in monthly reports, which is the basis of effective programme planning and monitoring for health managers.

Based on the multi-country evaluation of IMCI, which ran from 1999 to 2007, WHO released a position paper with a strong recommendation for health systems strengthening by ensuring the availability of skilled health workers, essential medicines, and supplies as a comprehensive package in all IMCI service delivery points [46]. The lack of comprehensive readiness, including essential equipment, drugs and logistics adversely affect the performance of the IMCI service provider and result in the poor quality of care [47, 48]. A global survey conducted by WHO found that the countries with a comprehensive implementation status of IMCI were 3.6 times were more likely to achieve MDG-4 target than other countries who could not adopt a comprehensive approach [27]. It is imperative to ensure comprehensive readiness of facilities for providing IMCI services, including pneumonia management, by learning lessons from this global evidence. We report substantial gaps regarding the overall readiness of public facilities in Bangladesh pertaining to pneumonia management. The readiness of private facilities regarding IMCI services is equally poor in Bangladesh [26]. Less than 1% of the private facilities were identified to be ready for providing child curative services. Only 20% of private facilities had an IMCI trained staffs. Without addressing these gaps, it will not be possible to adhere to the WHO quality of care standards related to the provision of care and experience of care [22]. The lack of comprehensive readiness, including essential equipment, drugs and logistics adversely affect the performance of the IMCI service provider and result in the poor quality of care [47, 48]. The readiness is much worse in lower-level facilities and facilities located in rural areas. The lower level facilities (UH&FWCs) are mostly located in rural areas and serve as the first point of contacts for the poorer and more disadvantaged sections of the population. Thus, the lower level of overall readiness in these facilities further contributes to the existing inequity in access to and utilisation of appropriate health services in Bangladesh and other low- and middle-income countries [2, 49–51]. According to Bangladesh Demographic Health Survey 2017–18, only 20% of the under 5 children with symptoms of acute respiratory infections sought care from public health facilities [52]. The poor level of readiness of public facilities may explain the low care-seeking practice from public health facilities.

Hypoxaemia is common among children with pneumonia and is one of the strongest predictors of mortality due to acute lower respiratory infections [53–55]. Integration of SpO2 assessment in existing IMCI services can significantly improve the accuracy of hypoxemia assessment and increase the validity (accuracy) of pneumonia classification (89, 96). In response to the need and global evidence, WHO recommended measuring SpO2 in routine IMCI services (23, 81). In addition, oxygen security is a vital concern in the context of the COVID-19 pandemic. The Government of Bangladesh adopted the WHO recommendation and decided to introduce pulse oximetry in IMCI services for SpO2 assessment [38]. Although the survey predates the Government of Bangladesh’s policy adoption regarding SpO2 assessment in IMCI services, it is helpful to understand the baseline situation of the country in this regard. Less than one-tenth of the facilities have pulse oximetry. The availability of pulse oximetry was not optimal even in referral hospitals as less than one-third of the DHs and one-fifth of the UHCs have readiness in this regard. This is similar to the status of many other low- and middle countries with limited resources [56]. To achieve the universal availability of pulse oximetry in all IMCI services contact points, the Government of Bangladesh needs to prioritise its procurement and efficiently manage its distribution. In addition to ensuring the availability of pulse oximetry, it is equally important to ensure the availability of oxygen in referral hospitals to aggressively correct and manage hypoxaemia [57, 58]. Unfortunately, one-third of the DHs and one-fourth of the UHCs do not have a reliable oxygen source which is essential for managing children with hypoxaemia. Effective oxygen therapy requires not only prompt and accurate detection of hypoxaemia but also appropriate administration of oxygen, combined with good clinical evaluation and management of the underlying condition. Bangladesh can learn from other low- and middle-income countries that have strategically invested in oxygen security in referral hospital and effectively reduced the burden of mortality due to pneumonia [59, 60]. Experience from four decades of ‘oxygen projects’ shows that effective improvement of oxygen systems in low-resource settings is possible but is complex and requires context-specific technical, clinical, and managerial solutions [61].

It is important to discuss some of the strengths and limitation of this study. BHFS-2017 was conducted with a nationally representative sample allowing us to generate specific estimates by facility types, locations and divisions. Moreover, the data collection tool used in BHFS-2017 was validated and adapted to the national context through expert consultations. The survey presents a snapshot of the service availability and readiness of the surveyed facilities since it only considered the availability of services and items on the day of the visit. Information regarding service availability is based on the self-reported status of the facility. Regarding readiness, the data collectors physically verified the availability of required and essential items and assessed their functionality where applicable. Although there were no globally validated criteria for assessing the service availability and readiness related to pneumonia management, we went through an expert consultation process under the leadership of IMCI programme in the Ministry of Health and Family Welfare to develop the service availability and readiness criteria for Bangladesh. The experts identified the lists of required and essential items as well as the scoring system based on consensus and under the leadership of the national IMCI programme. Although the lists and the scoring system are not validated externally, they reflect the national programme priorities. Lastly, we acknowledge that the gaps in service availability and readiness may be resulting from broader health systems building blocks issues such as health financing and governance. The availability of data did not allow us to explore and present such root causes, which needs to be explored through future research in this field.

## Conclusion

Our study findings reported low to moderate level of readiness for providing IMCI-based pneumonia management in public health facilities in Bangladesh. The readiness was significantly poorer among rural and lower-level facilities. Achieving the ambitious target of averting all preventable pneumonia-related mortality by 2025 will require effective coverage of pneumonia management with appropriate quality of care from the governments and supports from global level partners. Ensuring the service availability and readiness of health facilities is the first step towards promoting effective coverage and is integral to WHO’s quality of care framework. There are substantial gaps in the availability of IMCI services and readiness of essential items required for pneumonia management in public health facilities in Bangladesh. The situation is significantly worse in the lower level and rural facilities. Bangladesh should make substantial efforts to improve programme planning, implementation, and monitoring to address these critical gaps to ensure better provision of care for children suffering from pneumonia. Moreover, as IMCI has been scaled up in Bangladesh, there is a need for research on improving delivery strategies and overcoming barriers to implementation.

## Supplementary Information

**Additional file 1: Supplementary Table 1**: Definition of readiness for all pneumonia readiness items. **Supplementary Table 2**: List of core items required for pneumonia management services. **Supplement Figure 1**: Availability of core items required for pneumonia management services, BHFS 2017; presented in percentage.

## Data Availability

All BHFS data files are available from the MEASURE DHS database at http://www.dhsprogram.com/data/dataset_admin/download-datasets.cfm.

## Abbreviations

LMIC: Low- and Middle-Income Countries
WHO: World Health Organization
UNICEF: United Nations International Children’s Emergency Fund
GAPPD: Global Action Plan for Pneumonia and Diarrhoea
IMCI: Integrated Management of Childhood Illness
GoB: Government of Bangladesh
NIPORT: National Institute of Population Research and Training
icddr,b: International Centre for Diarrhoeal Disease Research, Bangladesh
ACPR: Associates for Community and Population Research
BHFS: Bangladesh Health Facility Survey
DH: District hospital
UHC: Upazilla Health Complex
MCWC: Maternal and Child Welfare Centre
UH&FWC: Union health and family welfare center
MoH&FW: Ministry of Health and Family Welfare
DGHS: Directorate general of health services
DGFP: Directorate general of family planning
NNHP&IMCI: National Newborn Health Programme and IMCI
