# Correction to: Managing pneumonia through facility-based integrated management of childhood management (IMCI) services: an analysis of the service availability and readiness among public health facilities in Bangladesh

**DOI:** 10.1186/s12913-021-06898-z

**Published:** 2021-08-26

**Authors:** Ahmed Ehsanur Rahman, Shema Mhajabin, David Dockrell, Harish Nair, Shams El Arifeen, Harry Campbell

**Affiliations:** 1 University of Edinburgh, Edinburgh, UK; 2 International Centre for Diarrhoeal Disease Research, Dhaka, Bangladesh

**Correction to: BMC Health Serv Res 21, 667 (2021)**

**https://doi.org/10.1186/s12913-021-06659-y**

Following publication of the original article [1], the authors identified an error in the family name of Ahmed Ehsanur Rahman.

The incorrect author name is: Ahmed Ehsanur Rahamn

The correct author name is: Ahmed Ehsanur Rahman

In addition, the corresponding email has been changed from eshanur@icddrb.org to ehsanur@icddrb.org.

The author group and corresponding email have been updated in this correction article and the original article [1] has been corrected.
