# Supplementary material for: Managing pneumonia through facility-based integrated management of childhood management (IMCI) services: an analysis of the service availability and readiness among public health facilities in Bangladesh
Source: BMC Health Serv Res. 2021 Jul 7;21:667. doi: 10.1186/s12913-021-06659-y (PMC8260350; doi:10.1186/s12913-021-06659-y)

*Supplementary Table 1: Definition of readiness for all pneumonia readiness items*

| Sl. | Items | Readiness definition |
| --- | --- | --- |
| 1. | IMCI trained staff | Availability of staff who received IMCI training ever |
| 2. | IMCI Chart booklet | Observed availability of IMCI chart booklet in the facility |
| 3. | Child weighing scale | Observed availability and functionality of child weighing scale in the facility |
| 4. | Infant weighing scale | Observed availability and functionality of infant weighing scale in the facility |
| 5. | ARI Timer or watch able to record seconds hand | Observed availability and functionality of ARI Timer or watch able to record seconds hand in the facility |
| 6. | Thermometer | Observed availability and functionality of thermometer in the facility |
| 7. | Pulse oximeter | Observed availability and functionality of pulse oxymeter in the facility |
| 8. | Amoxicillin DT or syrup | Observed availability of at least one valid Amoxicillin dispersible tablet or syrup in the facility |
| 9. | Inj Gentamycin | Observed availability of at least one valid injection gentamycin in the facility |
| 10. | Inj Diazepam | Observed availability of at least one valid injection Diazepam in the facility |
| 11. | Sulbutamol inhaler | Observed availability of at least one valid Sulbutamol inhaler in the facility |
| 12. | Sulbutamol syrup/tablet | Observed availability of at least one valid Sulbutamol syrup/tablet in the facility |
| 13. | Insulin syringe or disposable 5cc syringe | Observed availability of insulin syringe or disposable 5cc syringe in the facility |
| 14. | Spacers | Observed availability of spacers in the facility |
| 15. | IMCI register | Observed availability of IMCI register in the facility |
| 16. | IMCI referral form | Observed availability of IMCI referral form in the facility |

*Supplementary Table 2: List of core items required for pneumonia management services*

| Essential items | |
| --- | --- |
| i. | 1. IMCI trained staff |
| ii. | 3. CWS OR 4. IWS |
| iii. | 5. ARI Timer or watch able to record seconds hand |
| iv. | 6. Thermometer |
| v. | 8. Amoxicillin DT or syrup |
| vi. | 9. Inj Gentamycin |
| vii. | 10. Inj Diazepam |
| viii. | 11. & 12. Sulbutamol inhaler/syrup/tablet |
| ix. | 13. Syringe (5 cc) |
| x. | 15. IMCI register |

Supplement Figure 1: Availability of core items required for pneumonia management services, BHFS 2017; presented in percentage


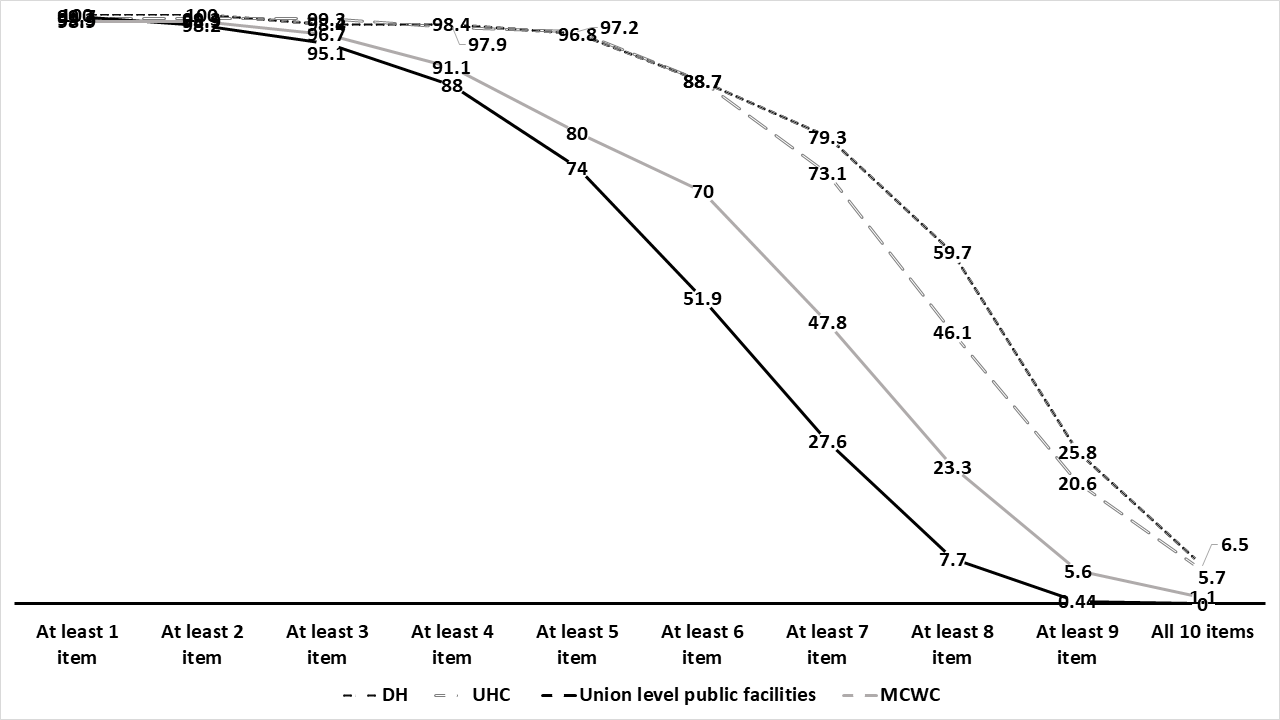

Supplement: Supplementary file 1 — Additional file 1: Supplementary Table 1: Definition of readiness for all pneumonia readiness items. Supplementary Table 2: List of core items required for pneumonia management services. Supplement Figure 1: Availability of core items required for pneumonia management services, BHFS 2017; presented in percentage. [file 12913_2021_6659_MOESM1_ESM.docx]
